# Posterior reversible encephalopathy syndrome after depletive lumbar puncture: a case report

**DOI:** 10.1186/1752-1947-8-261

**Published:** 2014-07-25

**Authors:** Michael Grelat, Jean-Baptiste Debaux, Jean-Louis Sautreaux

**Affiliations:** 1 Department of Neurosurgery, Bocage Central, University Hospital of Dijon, 14 rue Paul Gaffarel, Dijon 21000, France

**Keywords:** Lumbar puncture, Pathogenesis, Posterior reversible encephalopathy syndrome

## Abstract

**Introduction:**

Posterior reversible encephalopathy syndrome is a rare entity. Its pathophysiology is still poorly understood.

**Case presentation:**

We report the case of a 69-year-old White European woman who presented complete and proportional right hemiplegia, confusion, deviation of her head and eyes to the right, cortical blindness, and generalized tonic-clonic seizure 12 hours following a depletive lumbar puncture. Emergency cerebral magnetic resonance imaging showed bioccipital and left-side basal ganglia hyperintensities in the fluid attenuated inversion recovery and the diffusion-weighted images suggesting a radiological diagnosis of posterior reversible encephalopathy syndrome.

**Conclusions:**

The diagnosis is established on clinical and radiological signs. This is the first report of this kind in the literature. We present a case of posterior reversible encephalopathy syndrome after depletive lumbar puncture and we discuss the pathophysiology.

## Introduction

Posterior reversible encephalopathy syndrome (PRES) was first described by Hinchey in 1996 [1]. The initial term of leukoencephalopathy was later replaced by reversible posterior encephalopathy. This rare entity [2] corresponds to usually reversible vasogenic oedema in the posterior region of the brain, but is probably linked to a vasospasm of the posterior lobes of the brain.

In our case study, we discuss the pathophysiology of this syndrome.

## Case presentation

**Figure 1:**
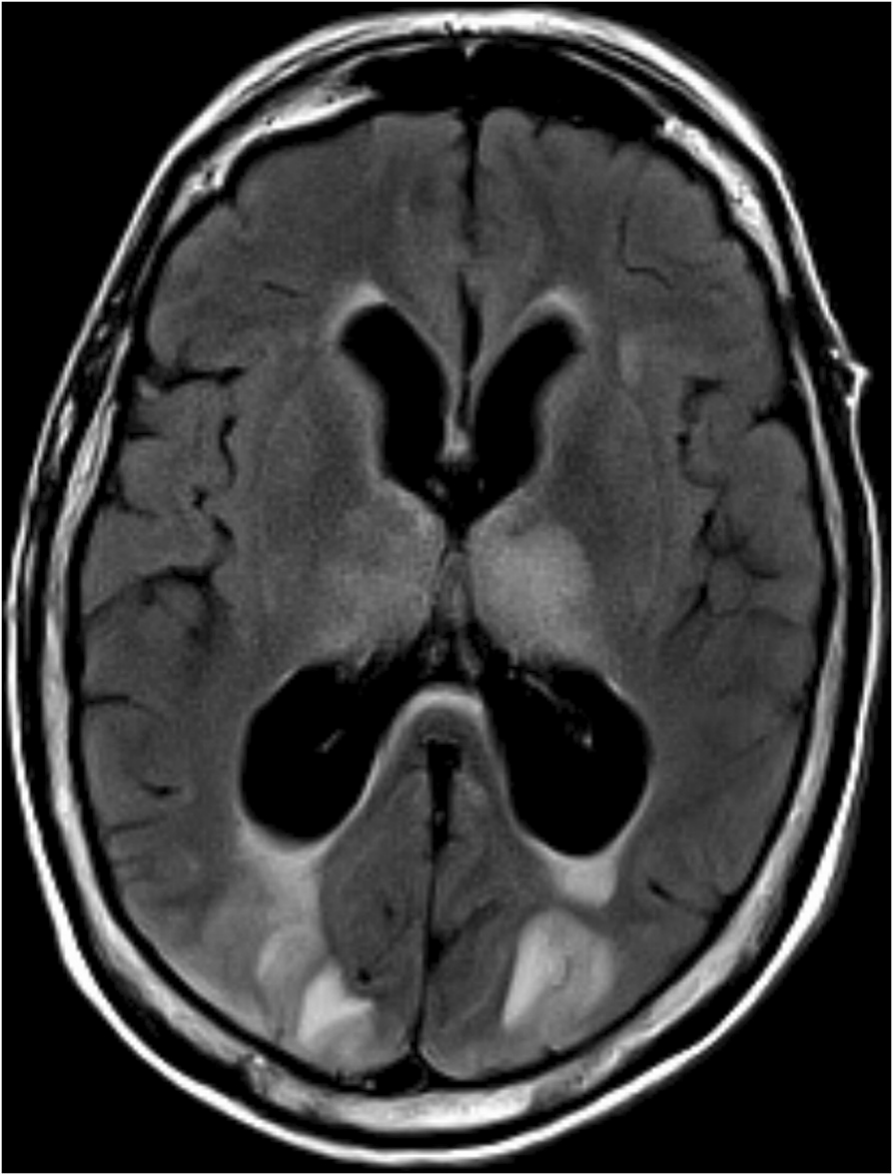
Brain axial fluid-attenuated inversion recovery weighted magnetic resonance image shows hyperintensity in the bioccipital region.

**Figure 2:**
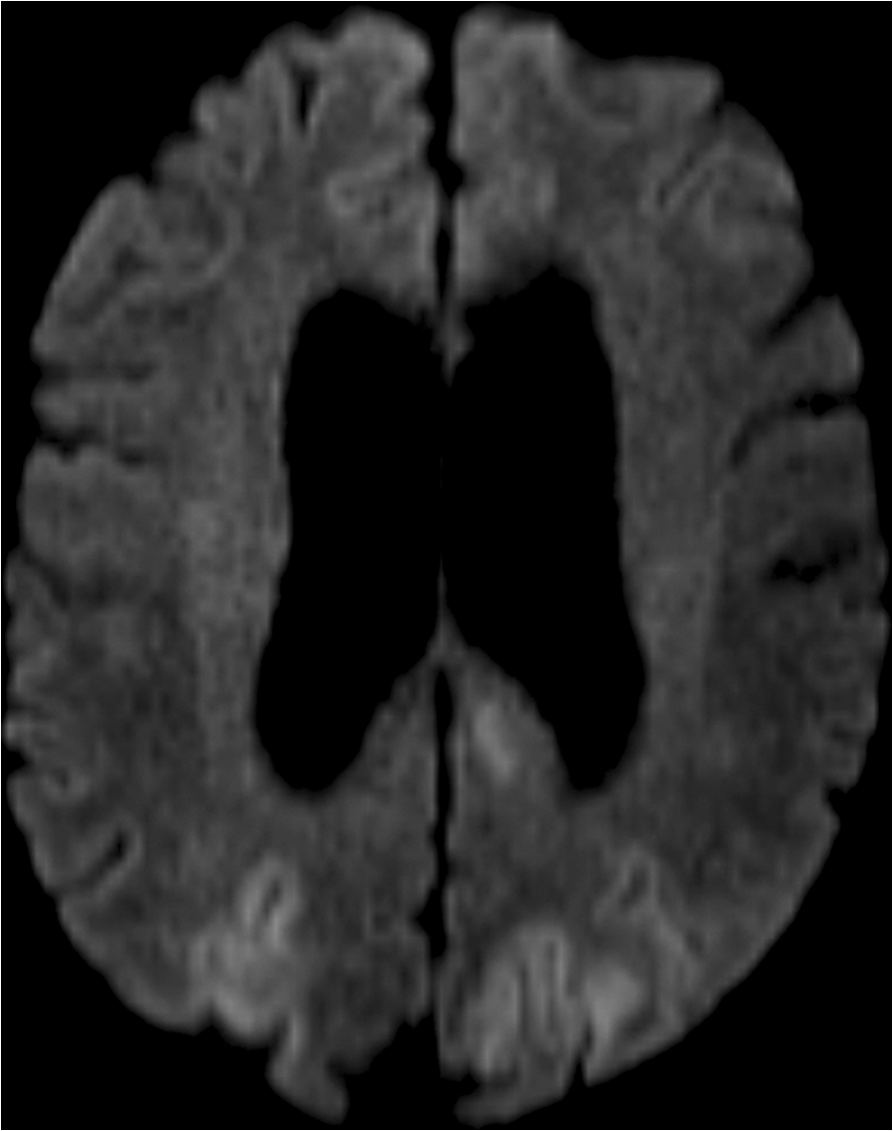
Brain axial diffusion-weighted magnetic resonance image with B1000 shows hyperintensity in the bioccipital region.

**Figure 3:**
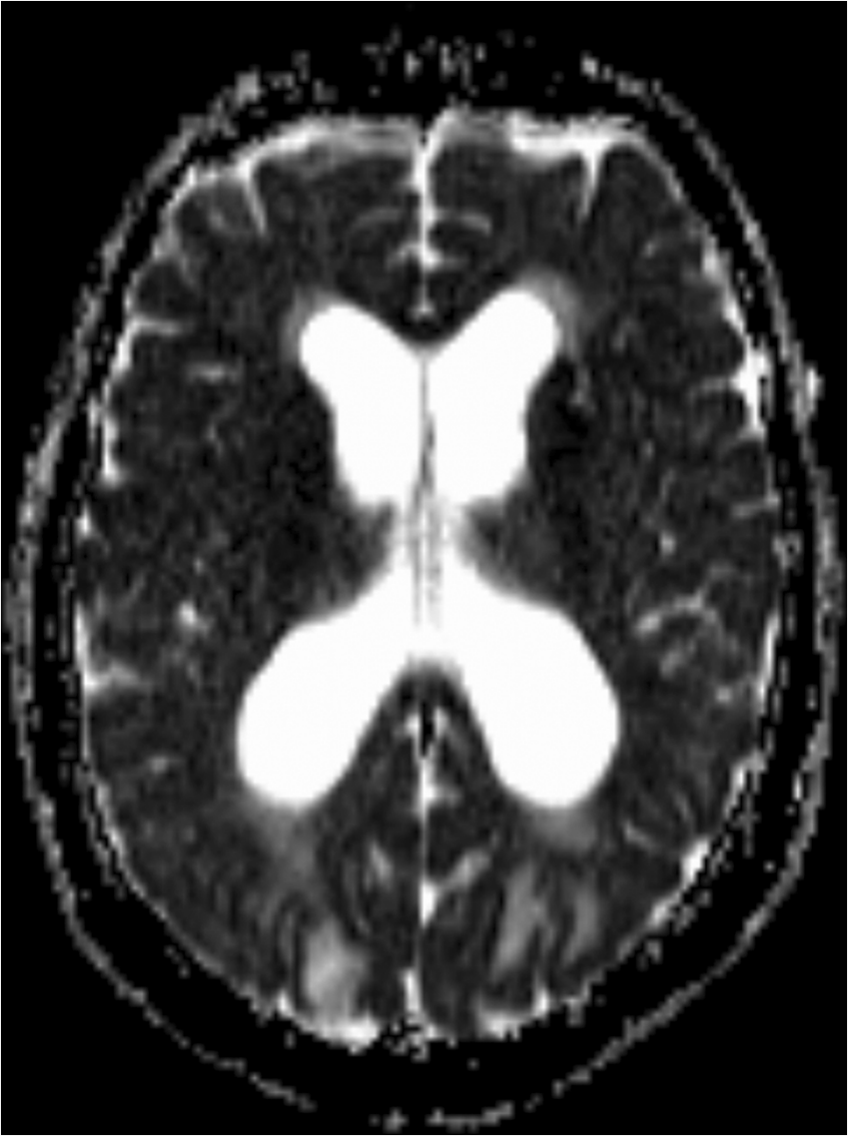
Brain axial apparent diffusion coefficient weighted magnetic resonance image shows hyperintensity in the bioccipital region.

A 69-year-old White European woman consulted with suspected chronic adult hydrocephalus. She had a history of subarachnoid haemorrhage from a ruptured aneurysm in the terminal region of her basilar artery and an unruptured aneurysm of her anterior communicating artery. She had undergone cerebral embolization 6 months earlier. On clinical examination she presented with gait disorders associated with cognitive impairment. Computed tomography found hydrocephalus. She was admitted to our neurosurgery unit for a depletive lumbar puncture (50cc). Twelve hours after the procedure, she experienced complete and proportional right hemiplegia, confusion, deviation of her head and eyes to the right, and cortical blindness. She also had a generalized tonic-clonic seizure which ceased with a dose of clonazepam. Her blood pressure at that time was normal. Emergency cerebral magnetic resonance imaging (MRI) showed bioccipital and left-side basal ganglia hyperintensities in the fluid attenuated inversion recovery and the diffusion-weighted images (Figures 1, 2 and 3). We observed a high apparent diffusion coefficient (ADC) suggesting vasogenic oedema. The above MRI image findings are consistent with a radiological diagnosis of PRES. We implemented antiepileptic therapy, calcium channel blockers to prevent vasospasm and antiplatelet therapy to prevent ischemia.

We noticed a pronounced improvement concerning her neurological deficit in the first 2 days and almost complete recovery of motor disorders after 5 days. Her vision problems persisted with minimal recovery of central vision. She also had massive amnesia.

After a positive progression for 5 days, she had perseverations and automatism with difficulty to make contact.

Electroencephalography showed posterior slow waves without any spikes.

Her evolution was successful with appropriate antiepileptic therapy. She left the unit to go to a rehabilitation centre. Four months later, her clinical state worsened and she went into a coma. A cerebral computed tomography scan showed acute hydrocephalus. An emergency ventriculoperitoneal shunt was performed. After the surgery, her state of consciousness returned to normal, but right hemiplegia appeared. The cerebral MRI showed a new PRES. The valve of the shunt was set to “high pressure” in order to reduce cerebrospinal fluid (CSF) flow. This reduced the hemiplegia but difficulties with executive functions remained.

## Discussion

PRES is a rare disease. On clinical examination, patients with PRES present headaches, altered mental functions, impaired alertness, drowsiness, stupor, seizures, focal neurological deficit and visual disturbances as well as visual hallucinations or cortical blindness [1-3]. As for imaging techniques, brain MRI is the gold standard. The main finding is vasogenic oedema, predominant in the posterior region in both sides, which is reversible in most cases [1,3,4]. It shows a high signal in T2, which is iso- or hypointense on T1, hyperintense and iso-intense B0 diffusion in B1000, a signal on elevated ADC [4]. The association of the subacute onset of clinical signs associated with radiological images gives the diagnosis of PRES.

Some promoting factors have been identified. These include renal failure, eclampsia, organ transplantation or bone marrow transplantation and immunosuppressive therapy [1]. Complications include cerebral ischemia, epilepsy and cerebral haemorrhage.

Two main theories are proposed to explain the pathophysiology. The most common is high blood pressure which causes cerebral hyperperfusion giving PRES predominantly in the posterior cerebral area [5,6]. The second hypothesis is vasoconstriction in the cerebral arteries causing cerebral hypoperfusion and inducing PRES [7].

A cytotoxic mechanism, such as the increase of inflammatory cytokines in preeclampsia, has also been described [7]. The direct action on endothelial cells increases endothelial permeability. Dysfunction of the blood–brain barrier plays a major role in PRES [8]. PRES probably has a multifactorial origin [1].

The patient in our case showed no high blood pressure spikes during her hospitalization. Neither did she have apparent post-lumbar puncture syndrome. We support the hypothesis of lumbar puncture induced PRES. In the literature there are a few cases of normotensive patients who have PRES after post-lumbar puncture syndrome [9], or caffeine intake for this syndrome [10]. In addition, some cases of PRES described with eclampsia occurred after an epidural breach of the dural sac [11]. Inadvertent perforation of the dura mater during an epidural procedure may contribute to the occurrence of PRES. CSF hypotension could weaken capillaries and the blood–brain barrier and could result in cerebral hypoperfusion leading to posterior cerebral vasoconstriction. Arutiunov *et al*. suggested that mechanical stimulation of the cerebral arteries could cause vasoconstriction [12]. This mechanical stimulation in our case could have been the decrease in ventricle size.

Regarding the two hypotheses, two different mechanisms could lead to the same clinical consequences: PRES [7,8]. Whatever the mechanism, PRES is the consequence of posterior cerebral vasospasm [2,9].

## Conclusions

PRES is a multifactorial entity. The diagnosis is established on clinical and radiological signs. In the present case, lumbar puncture and the subsequent CSF shunt may have been involved in this syndrome. Nonetheless, the pathophysiology and specific treatment are difficult to define.

## Consent

Written informed consent was obtained from the patient for publication of this case report and accompanying images. A copy of the written consent is available for review by the Editor-in-Chief of this journal.

## Abbreviations

ADC: Apparent diffusion coefficient; CSF: Cerebrospinal fluid; MRI: Magnetic resonance imaging; PRES: Posterior reversible encephalopathy syndrome.

## Competing interests

The authors have no competing interests to declare.

## Authors’ contributions

JBD and MG carried out the examination and monitored the patient. MG was a major contributor in writing the manuscript. J-L Sautreaux perform translation. All authors read and approved the final manuscript.
